# New distribution records of the savanna specialist fungus-farming ant *Cyatta* Sosa-Calvo et al. (Hymenoptera: Formicidae: Myrmicinae)

**DOI:** 10.3897/BDJ.4.e10673

**Published:** 2016-10-20

**Authors:** Aline Machado Oliveira, Rodrigo Machado Feitosa, Heraldo Luis Vasconcelos, Jonas Maravalhas

**Affiliations:** ‡ Universidade Federal do Paraná, Curitiba, Brazil; § Universidade Federal de Uberlândia, Uberlândia, Brazil

**Keywords:** Attini, range expansion, Neotropics, biogeography, Cerrado.

## Abstract

**Background:**

The fungus-farming ant genus *Cyatta* (Formicidae: Myrmicinae) is represented by a single species, *C. abscondita* Sosa-Calvo et al., known from a few localities in Brazil (in the states of Ceará, Minas Gerais, São Paulo and the Distrito Federal), and a single locality in the Misiones province, Argentina. *Cyatta* is known to occur predominantly in savanna habitats and occasionally in the transition zones between the Atlantic Forest and Cerrado.

**New information:**

The new records reported here significantly expand the previously known distribution of *Cyatta abscondita* and provide further support for the intimate relation between this species and the savannas of South America. We report the first occurrence of the genus in southern Brazil (Paraná state) and the westernmost occurrence (Bolivia) of *Cyatta abscondita*, which extend its distribution approximately 1450 km to the west. Finally, we discuss the importance of mapping inconspicuous species in order to develop strategies for protecting endangered areas and to increase our understanding of the evolutionary history of organisms and biomes.

## Introduction

The ant genus *Cyatta* is represented by a single species, *C. abscondita* which belongs to the Neoattini clade of fungus-farming ants. *Cyatta*, along with the monotypic genus *Kalathomyrmex* Klingenberg & Brandão, compose the sister group of all other genera in the Neoattini clade (Sosa-Calvo et al. 2013). This phylogenetic relationship is reflected on the etymology of the genus, where "*cy*" means sister (Brazilian Tupi language) and "*atta*" refers to the genus *Atta* Fabricius, the most derived representative of the fungus-farming ant clade. The specific epithet comes from Latin meaning "hidden” or “elusive" and refers to the difficulty in finding the species in the field (Sosa-Calvo et al. 2013).

**Figure 1.**
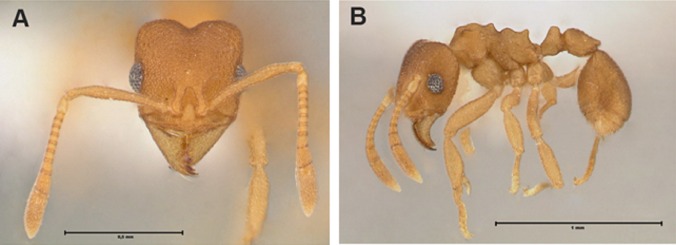
Worker of *Cyatta abscondita* from Crateús, Ceará State, Brazil. A: full-face view. B: lateral view. Photo by Chistiana Klingenberg.

Morphologically, *C. abscondita* can be recognized by the combination of the following characters: (i) worker and queen mandibles with four teeth; (ii) in ventral view, the metapleuron of workers and queens have two spiniform processes between the mid and hind coxae; (iii) apical margin of pygidium medially emarginate, "V"-shaped; and (iv) male forewing with or without a closed discal cell (1m-cu absent in Bolivian males, B. Boudinot pers. comm.). Workers are yellowish to light brown and relatively small, with less than 3 mm (Fig. 1) (Sosa-Calvo et al. 2013).

Observations on the biology of this species suggest that their colonies are relatively small, housing 20 to 26 workers (Sosa-Calvo et al. 2013). Nests can be established both in open or shaded areas with a very discrete hole in the ground, about 1 mm in diameter, used as an entrance and a nest depth ranging from 30 cm to 2 m. Workers are solitary foragers with their peak of activity after sunset, a behavior which may help to explain why the species is poorly represented in scientific collections. Like all the representative of the subtribe Attina (tribe Attini), this species cultivates a symbiotic fungus. Fungal cultivar of *Cyatta* are arranged in filamentous curtains suspended from the ceiling of the nest chambers, similar to what has been observed in *Kalathomyrmex emeryi* (Forel) and some species of *Mycocepurus* Forel (Fernández-Marín et al. 2004, Fernández‐Marín et al. 2005, Rabeling et al. 2007). Nothing is known about the reproductive biology of this species (Sosa-Calvo et al. 2013).

*Cyatta abscondita* was originally described based on specimens found in a Caatinga (xeric shrublands) area of northeastern Brazil (state of Ceará), Cerrado (tropical savanna) localities in central and southeastern Brazil (Distrito Federal, Minas Gerais and São Paulo), and transitional areas between the Cerrado and the Atlantic forest in the northern state of São Paulo (Sosa-Calvo et al. 2013). Recently, Ramos et al. (2016) reported the first occurrence of *Cyatta abscondita* outside the Brazilian borders, in a semi-deciduous forest in the northwestern region of Misiones province, Argentina.

Despite the records in Caatinga, and transitional localities of the Atlantic Forest in Brazil and Argentina, *C. abscondita* is predominantly an inhabitant of the Cerrado biome. The Brazilian Cerrado is the second largest biome in the country, accounting for about 23% of its territory, with approximately two million square kilometers (Bridgewater et al. 2004). Cerrado covers mainly the central states of Brazil and its southern limit is found in northern Paraná State, southern Brazil (Uhlmann et al. 1998).

In this paper we extend the known distribution of *Cyatta* based on new records from recent surveys in areas of Cerrado. We report the first record of the genus for southern Brazil and also the westernmost record, extending considerably its distribution to the western and southern boundaries of the Cerrado. This data reinforces the intimate relationship between *Cyatta* and the savannas and highlights the importance of protecting these endangered environments.

## Materials and methods

The new records of *C. abscondita* presented here come from surveys conducted within the ongoing project entitled “*Rede de Pesquisa Biota do cerrado* (*RPBcerrado 6*) - *Isoptera e Hymenoptera*” (project CNPq 457407/2012-3), coordinated by HLV. The project seeks to increase knowledge about the biodiversity of Hymenoptera and Isoptera in the Cerrado biome and identify the processes that generate and maintain their diversity in a biome highly threatened by human activities.

Samples were collected in 29 different localities of Cerrado, comprising the entire extension of the biome. In each collection site three transects of 400 m each were installed, separated by a distance of about 1 km (Vasconcelos et al. 2014). At every 20 m of the transect the nearest tree (height > 2.5 m) was marked and four arboreal pitfall traps were installed in different parts of the canopy. In addition, four pitfall traps were placed in the soil around the tree forming a 2 x 2 m grid with the tree trunk representing its center.

Ants were stored in 80% ethanol and processed in the *Laboratório de Ecologia de Insetos Sociais* of the *Universidade Federal de Uberlândia* (UFU). Vouchers are deposited in the *Museu de Biodiversidade do Cerrado* (MBC), Uberlândia, Minas Gerais; in the Padre Jesus Santiago Moure Entomological Collection (DZUP) of the *Universidade Federal do Paraná*, Curitiba, Paraná, Brazil; and in the *Museu de Zoologia da Universidade de São Paulo* (MZSP), São Paulo, SP, Brazil.

One of the sampling points of the project was the “Parque Estadual do Cerrado (hereinafter Cerrado State Park, or CSP), located near the town of Jaguariaíva, Paraná state (24°10'04,7"S, 49°40'05,5"W), at the southern boundary of the Cerrado biome in Brazil. Ant collections in the CSP were also conducted within the project “*Formigas (Hymenoptera, Formicidae) e a conservação dos campos naturais paranaenses: uma abordagem ecológica e taxonômica*” (project CNPq 459353/2014-4), coordinated by RMF. This project aims to sample the ant fauna of grasslands in the Paraná state following the sampling protocol mentioned above.

**Figure 2.**
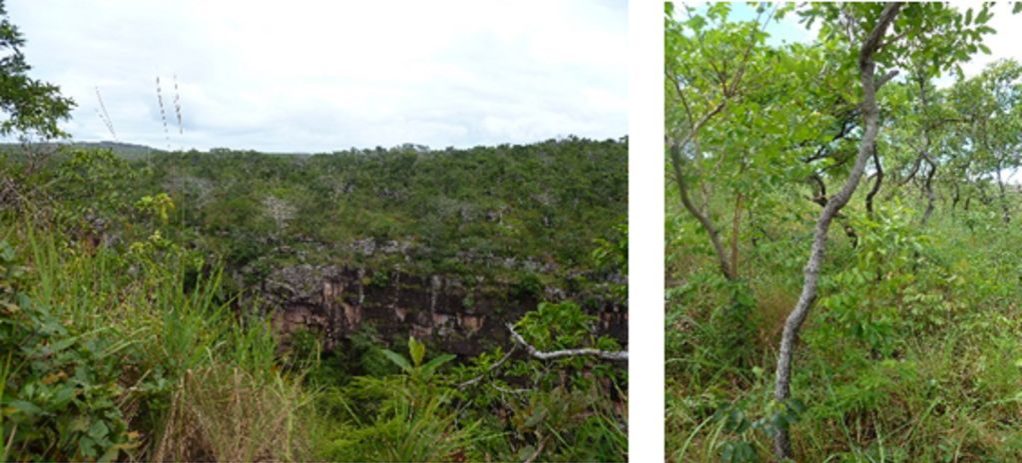
Parque Estadual da Serra de Ricardo Franco (*Serra Ricardo Franco* State Park, SRF), in Vila Bela da Santíssima Trindade, Mato Grosso state (14°55'07,3"S, 60°04'25,0"W). Photo by Jonas Maravalhas.

In addition to the CSP, new records of *Cyatta* come from four other Cerrado sites: Parque Estadual da Serra de Caldas (*Serra de Caldas* State Park, SCN), in Caldas Novas, Goiás (17°47'07,1"S, 48°40'07,5"W); Parque Estadual da Serra de Ricardo Franco (*Serra Ricardo Franco* State Park, SRF) (Fig. 2), in Vila Bela da Santíssima Trindade, Mato Grosso (14°55'07,3"S, 60°04'25,0"W); Estação Ecológica de Santa Bárbara (*Santa Barbara* Ecological Station, SBE), in Águas de Santa Bárbara, São Paulo (22°49'12,3"S, 49°13'07,3"W).

For all the aforementioned localities the predominant habitat is the Cerrado *sensu stricto*, an arboreal open woodland characterized by the presence of small trees with a canopy height of less than seven meters, shrubs, and abundant ground layer dominated by grasses (Gottsberger and Silberbauer-Gottsberger 2006). Cerrado soil is typically a red-yellow latosol, largely composed of well-drained and nutrient-poor quartz sand with a moderate clay content (Bramorski et al. 2012). The typical Cerrado climate is characterized by a marked dry season from May to September and mean annual temperature and precipitation of 23°C and ~1420 mm, respectively (Gottsberger and Silberbauer-Gottsberger 2006).

We also present here the first records of *Cyatta* from Cerrado areas of Bolivia, based on males collected at the Reserva Privada del Patrimonio Natural Potrerillos del Guenda (RNPG) in Santa Cruz de la Sierra (17°40' S, 63°27'W), and deposited in the University of California at Davis (UCDC) and at the Smithsonian Institution, Washington D.C (USNM).

The distribution map was generated using Quantum-Gis 2.12 based on the data from previous studies that recorded *C. abscondita* (Sosa-Calvo et al. 2013, Camacho and Vasconcelos 2015, Ramos et al. 2016), and our present results.

## Taxon treatments

### Cyatta abscondita

﻿Sosa﻿-Calvo et al. 2013

#### Materials

- **Type status:** Paratype. **Occurrence:** recordedBy: J. Sosa-Calvo, T.R. Schultz col.; individualCount: 5; sex: female; lifeStage: adult; **Taxon:** genus: Cyatta; specificEpithet: abscondita; **Location:** continent: South America; country: Brazil; stateProvince: Distrito Federal; county: Brasília; locality: Fazenda Água Limpa; verbatimElevation: 1071-1106m; locationRemarks: Cerrado sensu stricto; decimalLatitude: -15.9524; decimalLongitude: -47.90133; **Identification:** identifiedBy: J. Sosa-Calvo; dateIdentified: 2013; **Event:** samplingProtocol: Hand sampling; eventDate: 23.ii.2009-20.ix.2012; **Record Level:** collectionID: MZSP; collectionCode: USNMENT00756921-00758307
- **Type status:** Paratype. **Occurrence:** recordedBy: J. Sosa-Calvo, T.R. Schultz col.; individualCount: 2; sex: female; lifeStage: adult; **Taxon:** genus: Cyatta; specificEpithet: abscondita; **Location:** continent: South America; country: Brazil; stateProvince: Distrito Federal; county: Brasília; locality: Fazenda Água Limpa; verbatimElevation: 1071-1106m; locationRemarks: Cerrado sensu stricto; decimalLatitude: -15.9524; decimalLongitude: -47.90133; **Identification:** identifiedBy: J. Sosa-Calvo; dateIdentified: 2013; **Event:** samplingProtocol: Hand sampling; eventDate: 23.ii.2009-20.ix.2012; **Record Level:** collectionID: DZUP; collectionCode: USNMENT00758319-00758224
- **Type status:** Other material. **Occurrence:** recordedBy: Y. Quinet col.; individualCount: 1; sex: female; lifeStage: adult; **Taxon:** genus: Cyatta; specificEpithet: abscondita; **Location:** continent: South America; country: Brazil; stateProvince: Ceara; county: Cratéus; locality: RPPN Serra das Almas; verbatimElevation: 330m; locationRemarks: Caatinga; decimalLatitude: -5.16479; decimalLongitude: -40.67978; **Identification:** identifiedBy: R. Feitosa; dateIdentified: 2013; **Event:** samplingProtocol: Pitfall; eventDate: 20–30.iv.2003; **Record Level:** collectionID: MZSP
- **Type status:** Other material. **Occurrence:** recordedBy: J. Maravalhas col.; individualCount: 2; sex: female; lifeStage: adult; **Taxon:** genus: Cyatta; specificEpithet: abscondita; **Location:** continent: South America; country: Brazil; stateProvince: Distrito Federal; county: Brasília; locality: Reserva Ecológica IBGE; verbatimElevation: 1100m; locationRemarks: Cerrado sensu stricto; decimalLatitude: -15.85000; decimalLongitude: -48.05000; **Identification:** identifiedBy: J. Maravalhas; dateIdentified: 2013; **Event:** samplingProtocol: Pitfall; eventDate: 30.i.2008; **Record Level:** collectionID: MZSP
- **Type status:** Other material. **Occurrence:** recordedBy: A.A. Tavares col.; individualCount: 1; sex: female; lifeStage: adult; **Taxon:** genus: Cyatta; specificEpithet: abscondita; **Location:** continent: South America; country: Brazil; stateProvince: Minas Gerais; county: Paineiras; locality: Fazenda Olho D'Água; verbatimElevation: 611m; locationRemarks: Cerrado; decimalLatitude: -18.90628; decimalLongitude: -45.53527; **Identification:** identifiedBy: R. Feitosa; dateIdentified: 2013; **Event:** samplingProtocol: Winkler; eventDate: 22-24.v.1999; **Record Level:** collectionID: MZSP
- **Type status:** Other material. **Occurrence:** recordedBy: T.L.M. Frizzo col.; individualCount: 2; sex: female; lifeStage: adult; **Taxon:** genus: Cyatta; specificEpithet: abscondita; **Location:** continent: South America; country: Brazil; stateProvince: Minas Gerais; county: Paracatu; locality: RPPN Acangau; verbatimElevation: 671m; locationRemarks: Cerrado sensu stricto; decimalLatitude: -17.1790833; decimalLongitude: -47.0583056; **Identification:** identifiedBy: G. Camacho; dateIdentified: 2013; **Event:** samplingProtocol: Pitfall; eventDate: 12.iv.2011; **Record Level:** collectionID: MBC–UFU
- **Type status:** Other material. **Occurrence:** recordedBy: C. Rabeling & M. Bacci Jr col.; individualCount: 2; sex: female; lifeStage: adult; **Taxon:** genus: Cyatta; specificEpithet: abscondita; **Location:** continent: South America; country: Brazil; stateProvince: Săo Paulo; county: Itirapina; locality: Broa preserve; verbatimElevation: 530m; locationRemarks: Cerrado sensu stricto; decimalLatitude: -22.18517; decimalLongitude: -47.87754; **Identification:** identifiedBy: C. Rabelling; dateIdentified: 2013; **Event:** samplingProtocol: Hand sampling; eventDate: 21-28.vii.2011; **Record Level:** collectionID: MZSP; collectionCode: USNMENT00758220-00758217
- **Type status:** Other material. **Occurrence:** recordedBy: C. Rabeling & M. Bacci Jr col.; individualCount: 1; sex: male; lifeStage: adult; **Taxon:** genus: Cyatta; specificEpithet: abscondita; **Location:** continent: South America; country: Brazil; stateProvince: Săo Paulo; county: Itirapina; locality: Broa preserve; verbatimElevation: 530m; locationRemarks: Cerrado sensu stricto; decimalLatitude: -22.18517; decimalLongitude: -47.87754; **Identification:** identifiedBy: C. Rabelling; dateIdentified: 2013; **Event:** samplingProtocol: Hand sampling; eventDate: 21-28.vii.2011; **Record Level:** collectionID: MZSP; collectionCode: USNMENT00758204
- **Type status:** Other material. **Occurrence:** recordedBy: G.A. Castilho col.; individualCount: 1; sex: female; lifeStage: adult; **Taxon:** genus: Cyatta; specificEpithet: abscondita; **Location:** continent: South America; country: Brazil; stateProvince: Săo Paulo; county: Pindorama; locality: Fazenda Águas Claras; verbatimElevation: 494m; locationRemarks: Floresta Estacional Semidecidual; decimalLatitude: -21.4023; decimalLongitude: -48.6873; **Identification:** identifiedBy: R. Feitosa; dateIdentified: 2013; **Event:** samplingProtocol: Pitfall; eventDate: 16.viii.2011; **Record Level:** collectionID: MZSP
- **Type status:** Other material. **Occurrence:** recordedBy: G.A. Castilho col.; individualCount: 1; sex: female; lifeStage: adult; **Taxon:** genus: Cyatta; specificEpithet: abscondita; **Location:** continent: South America; country: Brazil; stateProvince: Săo Paulo; county: Sales; locality: Estaçăo Experimental; verbatimElevation: 414m; locationRemarks: Floresta Estacional Semidecidual; decimalLatitude: -21.5222; decimalLongitude: -49.3013; **Identification:** identifiedBy: R. Feitosa; dateIdentified: 2013; **Event:** samplingProtocol: Pitfall; eventDate: 17.viii.2011; **Record Level:** collectionID: MZSP
- **Type status:** Other material. **Occurrence:** recordedBy: A.M. Oliveira, R.Feitosa, J. Maravalhas, H. Vasconcelos col.; individualCount: 5; sex: female; lifeStage: adult; **Taxon:** genus: Cyatta; specificEpithet: abscondita; **Location:** continent: South America; country: Brazil; stateProvince: Paraná; county: Jaguariaíva; locality: Parque Estadual do Cerrado; verbatimElevation: 899m; locationRemarks: Cerrado sensu stricto; decimalLatitude: -24.17972; decimalLongitude: -49.6680; **Identification:** identifiedBy: R. Feitosa; dateIdentified: 2015; **Event:** samplingProtocol: Pitfall; eventDate: 15.i.2015; fieldNumber: T64p15-T66p6; **Record Level:** collectionID: DZUP
- **Type status:** Other material. **Occurrence:** recordedBy: H.L. Vasconcelos and T.L.M. Frizzo; individualCount: 1; sex: female; lifeStage: adult; **Taxon:** genus: Cyatta; specificEpithet: abscondita; **Location:** continent: South America; country: Brazil; stateProvince: Goias; county: Caldas Novas; locality: Parque Estadual Serra de Caldas Novas; verbatimElevation: 952m; locationRemarks: Cerrado; decimalLatitude: -17.7852; decimalLongitude: -48.6686; **Identification:** identifiedBy: J. Maravalhas; dateIdentified: 2015; **Event:** samplingProtocol: Pitfall; eventDate: 11.i.2011; fieldNumber: T31; **Record Level:** collectionID: LEIS-UFU
- **Type status:** Other material. **Occurrence:** recordedBy: H.L. Vasconcelos, Jonas B. Maravlhas & Elmo B. A. Koch col; individualCount: 2; sex: female; lifeStage: adult; **Taxon:** genus: Cyatta; specificEpithet: abscondita; **Location:** continent: South America; country: Brazil; stateProvince: Mato Grosso; county: Vila Bela da Santíssima Trindade; locality: Parque Estadual Serra de Ricardo Franco; verbatimElevation: 648m; locationRemarks: Cerrado; decimalLatitude: -14.91861; decimalLongitude: -60.0736; **Identification:** identifiedBy: J. Maravalhas; dateIdentified: 2015; **Event:** samplingProtocol: Pitfall; eventDate: 02.vii.2014; fieldNumber: T60; **Record Level:** collectionID: LEIS-UFU
- **Type status:** Other material. **Occurrence:** recordedBy: H.L. Vasconcelos, Jonas B. Maravlhas & Elmo B. A. Koch col; individualCount: 2; sex: female; lifeStage: adult; **Taxon:** genus: Cyatta; specificEpithet: abscondita; **Location:** continent: South America; country: Brazil; stateProvince: Săo Paulo; county: Águas de Santa Barbara; locality: Estaçăo Ecológica Águas de Santa Barbara; verbatimElevation: 643m; locationRemarks: Cerrado; decimalLatitude: -22.8200; decimalLongitude: -49.2186; **Identification:** identifiedBy: J. Maravalhas; dateIdentified: 2015; **Event:** samplingProtocol: Pitfall; eventDate: 12..ix2014; fieldNumber: T61; **Record Level:** collectionID: LEIS-UFU
- **Type status:** Other material. **Occurrence:** recordedBy: Cline & Wappes; individualCount: 3; sex: male; lifeStage: adult; **Taxon:** genus: Cyatta; specificEpithet: abscondita; **Location:** continent: South America; country: Bolivia; stateProvince: Santa Cruz Department; county: Santa Cruz de la Sierra; locality: Reserva Natural Potrerrilos del Guenda; verbatimLocality: 37m; locationRemarks: Amazon Forest/Cerrado; decimalLatitude: -17.66666; decimalLongitude: -63.45000; **Identification:** identifiedBy: B. Boudinot; **Event:** eventDate: 12-13.x.2007; **Record Level:** collectionID: UCDC
- **Type status:** Other material. **Occurrence:** recordedBy: Wappes & Morris; individualCount: 1; sex: male; lifeStage: adult; **Taxon:** genus: Cyatta; specificEpithet: abscondita; **Location:** continent: South America; country: Bolivia; stateProvince: Santa Cruz Department; county: Santa Cruz de la Sierra; locality: Reserva Natural Potrerillos del Guenda; verbatimElevation: 37m; locationRemarks: Amazon Forest/Cerrado; decimalLatitude: -17.66666; decimalLongitude: -63.45000; **Identification:** identifiedBy: B. Boudinot; **Event:** eventDate: 14.x.2007; **Record Level:** collectionID: UCDC
- **Type status:** Other material. **Occurrence:** recordedBy: Wappes & Morris; individualCount: 1; sex: male; lifeStage: adult; **Taxon:** genus: Cyatta; specificEpithet: abscondita; **Location:** continent: South America; country: Bolivia; stateProvince: Santa Cruz Department; county: Santa Cruz de la Sierra; locality: Reserva Natural Potrerillos del Guenda; verbatimElevation: 37m; locationRemarks: Amazon Forest/Cerrado; decimalLatitude: -17.00000; decimalLongitude: -63.45000; **Identification:** identifiedBy: Jeffrey Sosa-Calvo; **Event:** eventDate: 14.x.2007; **Record Level:** collectionID: USNM

#### Taxon discussion

The workers examined were all identified as *Cyatta abscondita* (Fig. 1), since they match each of the diagnostic characters of the species (Sosa-Calvo et al. 2013)

## Discussion

Five new records of this rarely collected ant genus were added to the nine previously known, totaling 14 localities where *Cyatta abscondita* has been found, raising by more than 50% the number of occurrence sites for *Cyatta*. We report 15 specimens for the new occurrence sites, five in CSP, five in RNPG, two in SBE, two in SRF and one in SCN. All the newly collected specimens were captured in pitfall traps placed on the ground, except by the Bolivian specimens (RNPG) for which the collection method is unknown. The fact that such a small amount of workers was sampled from 6,960 pitfall traps installed in the soil of the 29 Cerrado localities of the project highlights the elusive nature of this species.

**Figure 3.**
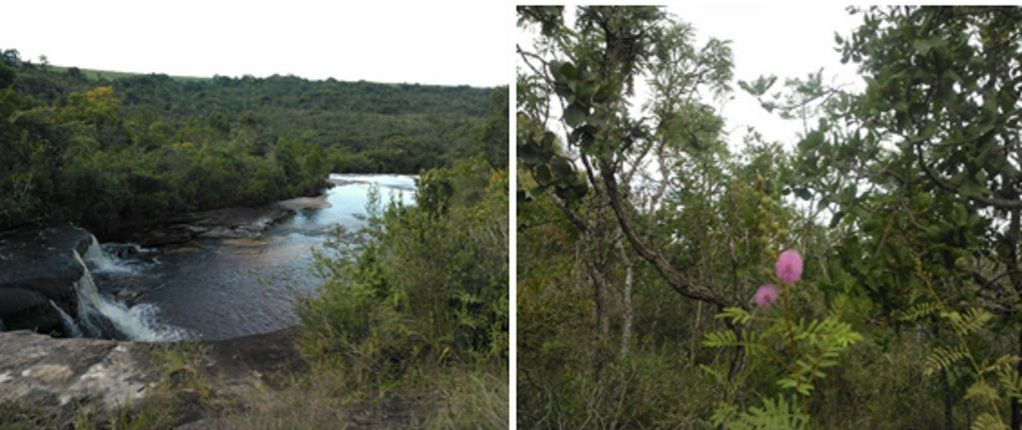
Parque Estadual do Cerrado (Cerrado State Park, CSP), in Jaguariaíva, Paraná state (24°10'04,7"S, 49°40'05,5"W). Photo by Rodrigo Feitosa.

The southernmost record for this genus is the province of Misiones, Argentina (Ramos et al. 2016); however, the record presented here for the CSP in Jaguariaíva, Paraná, represents the first record of *Cyatta* for southern Brazil (Fig. 3). We emphasize the importance of this record in the Cerrado State Park, which carries an inestimable importance for biodiversity conservation in the region. This park represents the southern limit of the Cerrado, and is the only fragment of this biome legally protected in southern Brazil (Linsingen et al. 2006).

The westernmost limit of the genus distribution was previously represented by specimens collected in the municipality of Sales, São Paulo state. The males collected in RNPG, Santa Cruz de la Sierra, Bolivia, extend the western limit of the known distribution by an impressive 1450 kilometers.

**Figure 4.**
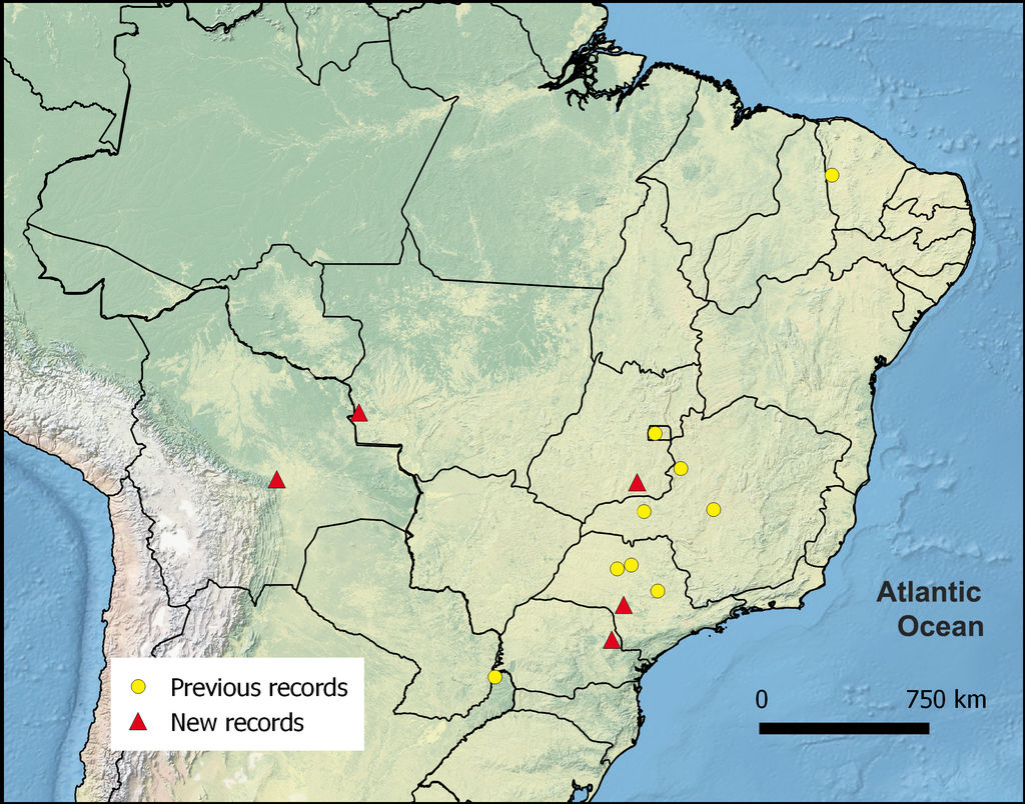
Distribution map for *Cyatta abscondita*.

All the sites where *Cyatta* was collected are savannas or transition zones between semi-deciduous Atlantic Forest and Cerrado (Table 1). These new records not only corroborate previous observations regarding the distribution of *C. abscondita* in savannas, but broaden its potential distribution over most of this biome. Although it was recently collected in a subtropical semi-deciduous native forest in Argentina (Ramos et al. 2016), it seems reasonable to assume that *Cyatta abscondita* is an inconspicuous though broadly distributed element of the so called South American dry diagonal (Fig. 4), which includes the savanna lands of northeastern and central Brazil (Caatinga and Cerrado, respectively), and the Chaco areas of Paraguay and Argentina (Prado and Gibbs 1993).

It is essential to improve our knowledge about the natural history and geographic distribution of life forms to develop relevant conservation strategies for ants (Guenard et al. 2012). This is especially true in ecosystems were the presence of endemic species is threatened due to the rapid loss of habitat resulting from unsustainable human exploitation, i.e. the biodiversity hotspots (Myers et al. 2000). The Brazilian Cerrado is one such hotspot, with more than four thousand species of endemic plants and more than one hundred species of endemic vertebrates (Myers et al. 2000). Over 60% of the original Cerrado distribution is still covered by natural vegetation, but in an asymmetrical fashion, with most remnants located in the northern region (Sano et al. 2010). Unfortunately, only about 3.1% of the original Cerrado vegetation is under strict environmental protection (M.M.A 2016), a situation for concern.

Another biome of occurrence for *Cyatta*, the Brazilian Caatinga, is even less protected than the Cerrado (Silva et al. 2004). The discovery of *Cyatta abscondita* in Cerrado areas of western Brazil and Bolivia, suggests that this species may also occur in poorly explored habitats of South America, as the Chaco, for example (Bucher 1982). Our study suggests that increasing the search for elusive and inconspicuous species may lead to discoveries that can alter our fundamental understanding of the evolutionary history of organisms and biomes.

## Supplementary Material

XML Treatment for Cyatta abscondita

## Figures and Tables

**Table 1.** Distribution records for *Cyatta abscondita*.

**Municipality**	**State/Country**	**Political Region**	**Coordinates**	**Biome**	**Source**
**Previous records**					
Crateús	Ceará (CE)/Brazil	Northeast	S 05.1647°, W 40.6797°	Caatinga	Sosa-Calvo et al. 2013
Brasília	Distrito Federal (DF)/Brazil	Midwest	S 15.8500°, W 48.0500°	Cerrado	Sosa-Calvo et al. 2013 ﻿
Paineiras	Minas Gerais (MG)/Brazil	Southeastern	S 18.9062°, W 45.5352°	Cerrado	Sosa-Calvo et al. 2013
Paracatu	Minas Gerais (MG)/Brazil	Southeastern	S 17.1790°, W 47.0583°	Cerrado	Sosa-Calvo et al. 2013
Uberlândia	Minas Gerais (MG)/Brazil	Southeastern	S 19° 10’, W 48° 24’	Cerrado	Camacho and Vasconcelos 2015
Itirapina	São Paulo (SP)/Brazil	Southeastern	S 22.18517°, W 47.87754°	Cerrado	Sosa-Calvo et al. 2013
Pindorama	São Paulo (SP)/Brazil	Southeastern	S 21.4023°, W 48.6873°	Atlantic Forest-Cerrado	Sosa-Calvo et al. 2013
Sales	São Paulo (SP)/Brazil	Southeastern	S 21.5222°, W 49.3013°	Atlantic Forest-Cerrado	Sosa-Calvo et al. 2013
Puerto Iguazu	Misiones/Argentina	Mesopotamia	S 25°41'23", W 54°27'30"	Atlantic Forest	Ramos et al. 2016
**New records**					
Jaguariaíva	Paraná (PR)/Brazil	South	S 24°10'04,7", W 49°40'05,5"	Cerrado	Present study
Caldas Novas	Goiás (GO)/Brazil	Midwest	S 17°47'07,1", W 48°40'07,5"	Cerrado	Present study
Vila Bela da Santíssima Trindade	Mato Grosso (MT)/Brazil	Midwest	S 14°55'07,3", W 60°04'25,0"	Cerrado	Present study
Águas de Santa Barbara	São Paulo (SP)/Brazil	Southeast	S 22°49'12,3", W 49°13'07,3"	Cerrado	Present study
Santa Cruz de la Sierra	Santa Cruz/Bolivia	Santa Cruz	S 17°40', W 63°27'	Cerrado	Present study
